# A comparative review of three cloud-based platforms for microbial whole genome sequencing analysis

**DOI:** 10.1017/ash.2026.10316

**Published:** 2026-04-06

**Authors:** Munok Hwang, Hosoon Choi, Piyali Chatterjee, Chetan Jinadatha

**Affiliations:** 1 Research Service, Central Texas Veterans Health Care System, Temple, USA; 2 Infectious Diseases Division, Central Texas Veterans Health Care System, Temple, USA; 3 College of Medicine, Texas A & M University, Bryan, USA

## Abstract

**Objective:**

Whole genome sequencing (WGS) of microbes is a powerful tool for pathogen detection, antimicrobial resistance (AMR) genes, and outbreak analysis. However, WGS data analysis is a challenging process and a major limitation of adopting WGS in clinical use. In this manuscript, we performed in-depth review of three cloud-based sequencing data analysis platforms that are user-friendly and require minimal or no bioinformatics expertise.

**Methods:**

Total of 90 bacterial isolates collected from inpatient units of the Central Texas Veterans healthcare system were sequenced on Nextseq 550 system (Illumina). FASTQ files generated after the sequencing were uploaded to the sequence analysis platforms: BugSeq™, EPISEQ® CS, and Solu. Each platform’ s usability, cost, output type, turnaround time from uploading files to results, and analysis output including number of identifiable pathogen species, AMR genes, and epidemiological clusters were assessed.

**Results:**

All platforms were easy to use and offered analysis features including pathogen identification, AMR gene and plasmid detection, and epidemiological analysis. Analysis results were generated within hours (BugSeq™ & EPISEQ® CS, 3 ∼ 4 hrs; Solu, 1 hr) after sequence file upload. All platforms identified most pathogen species correctly (BugSeq™, 89; EPISEQ® CS, 83; Solu, 87). Among 90 isolates, wide-ranging number of AMR genes were detected (BugSeq™, 83; EPISEQ® CS, 358; Solu, 137). Epidemiological clusters (closely related isolates) detection was also varied (BugSeq™, 9; EPISEQ® CS, 11; Solu, 12) because each platform used different methodologies.

**Conclusion:**

While users need to consider factors such as cost, IT infrastructure, and data security, cloud-based platforms may ease barriers for adopting WGS for clinical and infection prevention purposes.

## Introduction

Whole genome sequencing (WGS) of microbes is a powerful tool for rapid and accurate detection of pathogens and can be utilized in infection prevention and control (IPC) for surveillance and outbreak analysis.^1,2^ Whole genome-based surveillance enables the identification of genetic relationships among pathogens and facilitates tracking the source of outbreaks.^3^ Pathogens can be identified directly from specimens without the need for traditional bacterial culture.^4^ With high-quality WGS data and appropriate analysis, comprehensive information can be obtained, including pathogen identification, bacterial strain typing, clonal relationships between pathogens, and detection of antimicrobial resistance (AMR) genes.^2^ Recent advancements in WGS platforms have simplified sequencing processes therefore reducing sequencing costs, which in turn has led to many clinical laboratories adopting WGS for routine clinical use and outbreak investigations.^5^ However, easy-to-use comprehensive bioinformatic platforms remain limited and often pose a barrier to the widespread application of WGS. Data analysis is a complex and challenging process that typically requires bioinformatics expertise, coding knowledge, and significant computing power. Although sequencing costs have decreased and technology has advanced, user-friendly sequencing data analysis platforms that provide fast and accurate information without requiring in-depth bioinformatics knowledge or substantial computing resources are still lacking. This remains a major limitation to the more widespread adoption of WGS, particularly for routine clinical and IPC use. In this manuscript, we provide an in-depth review of three commercially available cloud-based sequencing data analysis platforms: BugSeq^TM^ ^6^, EPISEQ® CS, and Solu^7^ that require minimal to no bioinformatics expertise to utilize WGS data and perform complex analysis.

## Methods

### Bacterial sample collection

Bacterial samples were collected from the inpatient units at the Central Texas Veterans Healthcare System (CTVHCS) from August 2024 to March 2025. The clinical lab isolated pathogens from the specimens and bacterial species were identified with MALDI-TOF (Matrix-Assisted Laser Desorption/Ionization-Time-Of-Flight) (BioMérieux). After MALDI-TOF, the bacterial isolates were subject to WGS. The information for the isolates’ specimen type, hospital unit, and collection date is listed on the supplement data.

### Whole genome sequencing and analysis

A total of 90 bacterial isolates were freshly cultured overnight prior to genomic DNA extraction. Genomic DNA was extracted using QIAmp Micro kit (Qiagen), and quality was assessed using a NanoDrop spectrophotometer and a Qubit 2.0 Fluorometer with 1x ds HS DNA kit (Thermo Fisher). Library preparation was performed using the Illumina DNA Prep Kit, and sequencing was conducted on the Illumina NextSeq 550 system using the NextSeq 550 Mid Output Kit for 150 bp paired end reads. FASTQ files generated via Local Run Manager (Illumina) were uploaded to the three sequence analysis platforms: BugSeq™ (https://bugseq.com/), EPISEQ® CS (https://login.biomerieux.com/episeq/), and Solu (https://www.solugenomics.com/). The same test data set with median coverage depth 113.9X (supplement data) was used for all the platforms. We assessed the platforms’ general features including usability, turnaround time to analysis output, output types, available analysis tools, and cost per sample. For performance, we reviewed the detected pathogen species, number of identified AMR gene types, and epidemiological clusters. pubMLST (https://pubmlst.org/) was used as reference tool to identify bacterial species using WGS data. We also reached out to vendor and summarized the methodologies employed by each platform to explain any observed differences or discrepancies. No statistical analysis was carried out. The CTVHCS Institutional Review Board approved this study.

## Results

### Features assessment

**Figure 1.**
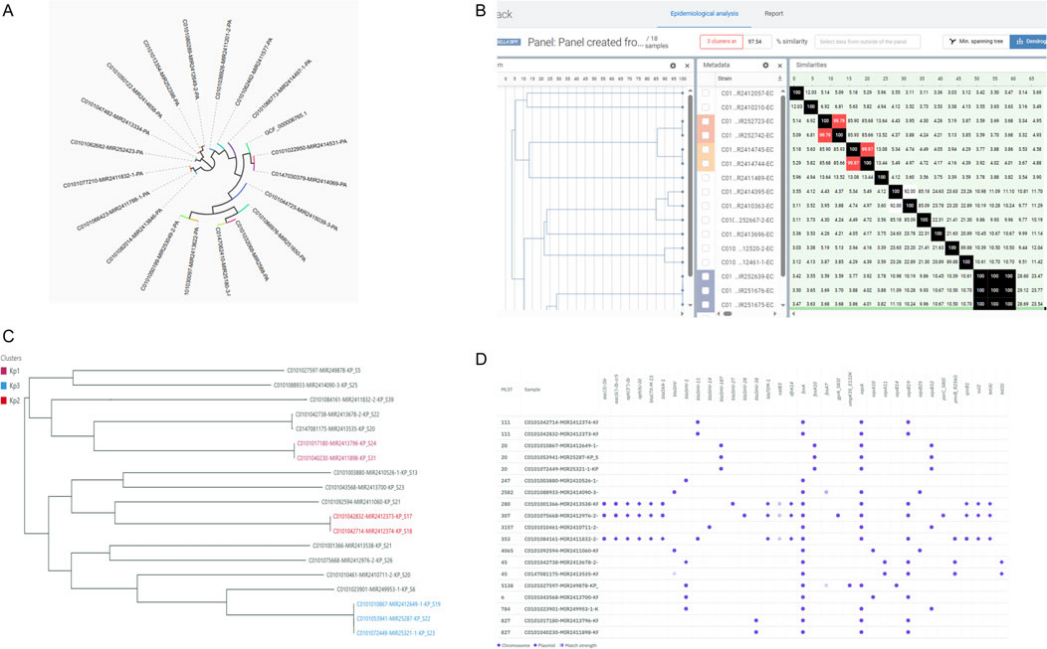
Output from three platforms. A. Dendrogram screenshot of *P. aeruginosa* from BugSeq^TM^. B. Panel epidemiology screen shot of *E. coli* from EPISEQ® CS. C. Exported phylogenetic tree picture of *K. pneumoniae* collection from Solu. D. Exported resistome heatmap picture of *K. pneumoniae* collection from Solu.

The graphic user interfaces of three platforms were intuitive and allowed users to easily navigate from uploading FASTQ files to reviewing metrics as well as output results. Although there were subtle differences in features, all the platforms offered similar standard analysis, including sequence read quality assessment, *de novo* assembly, pathogen identification and strain typing, AMR gene and virulence factor detection, plasmid detection, and epidemiological analysis. Table 1 summarizes the key features of each platform, and Figure 1 shows an example of a phylogenetic tree along with AMR resistome outputs from each platform.

**Table 1.** Comparison of the software features

Features	Bugseq^TM^	EPISEQ® CS	Solu
Input format	FASTQ and BAM	FASTQ and FASTA	FASTQ and FASTA
*De novo* assembly	Illumina, ONT, PacBio, Ion Torrent	Illumina only (SPAdes)	Illumina (Shovill) and ONT (Dragonflye)
Quality assessment	FastQC, QUAST statistics, BUSCO	EPISEQ® CS QC	FastQC
Species identification	Bacteria and virus (BugSplit)	Selected 14 species of bacteria (EPISEQ® CS database)	Bacteria and fungi (BactoInspector)
Sequence typing	PubMLST	Whole-genome MLST (wgMLST)	PubMLST
AMR resistome	Curated database Predict Drug resistance with confidence reporting	Curated databases encompass 4 public databases	AMRFinderPlus
Virulence detection	Curated database (detects virulence only for *C. diff, Staph aureus* and *E. coli* with their own curated database)	EPISEQ® CS virulome database	Virulence Factor Database
Plasmid detection	MOB-suite	EPISEQ® CS plasmid functionality	MOB-suite
Outbreak analysis	refMLST, assigned cluster addresses. Phylogenetic tree (circular) and clustering based on allele distance matrix	wgMLST based phylogenetic analysis. Phylogenetic tree and clustering based on the similarity distance	Reference based and reference-free alignment. Phylogenetic tree and clustering based on pairwise SNP distance
Sample selection	Lab	Panel	Collection
Output files	FASTA, .zip file, CSV (AMR, plasmid, QC, typing, distance matrix) Html (all and per sample report), PDF (quality asses)	FASTA, PDF (report for individual sample and panel including dendrogram, QC, and typing), Excel (resistome, similarity distance)	FASTA (genome, plasmid), .png (tree, resistome heatmap), Excel (AMR, plasmid detection, MLST, SNP cluster, SNP distance)
Run time	3 hrs for 28 samples, outbreak analysis took longer	Mostly 3 ~ 4 hrs, sometimes longer	~10 min for one sample, 1 hr for 50 samples.
Cost per sample*	$~$$	$	$
Unique features	Metagenomic classification	Epidemiological analysis	Public genome comparison

* Since pricing is proprietary, we used a symbolic scale to indicate cost ranges per sample: $ = approximately $10–20, $$ = approximately $20–40, $$$ = greater than $40.

#### BugSeq^TM^

*Usability*: Users are required to choose sequencing platform, sample type, sequenced materials, and selection options for Lab designation, metagenomic database, and outbreak analysis prior to uploading files. Files can be uploaded directly or imported from BaseSpace, a subscription-based online storage platform used with Illumina WGS equipment, or from Sequence Read Archive (SRA), a free online repository provided by the National Institutes of Health. Analysis results were generated for each submission, and BugSeq^TM^ notifies users via email upon completion of analysis. The main menu includes options for new analysis, previous analyses, data exploration (insights and clustering), Lab name, and documentation. The “Previous Analysis” page displays all analysis names with clickable buttons to access results. Each analysis includes summary reports for all samples, per-sample reports, outbreak analysis, and metagenomic classification. A minimum purchase of 25 samples is required, with lower per-sample pricing available for higher volumes. Subscription credits expire after six months.

*Analysis features*: The summary report includes an interactive html overview page and a downloadable PDF, which can be shared with other members if a multi-user lab profile is created. BugSeq^TM^ provides read and assembly statistics table, including median coverage depth which are required information for sequence submission to databases like GenBank. Per-sample reports include confidence levels for AMR gene detection, virulence factors, and plasmid identification. All results can be exported as a .csv file, consolidated into a single worksheet for all submitted species. Users can also copy result tables, export plots, and configure table columns. For outbreak analysis, users must create a “Lab” to group samples. Results include a circular phylogenetic tree and cluster assignments based on inter-genome allele distances. A distance matrix with cluster addresses per sample is downloadable; however, users can’t download the dendrogram as a file and need to take a screenshot for publication or reporting (Figure 1A). A unique feature of BugSeq^TM^ is metagenomic classification, which displays the taxonomic composition of each sample. However, the assembled FASTA file format doesn’t fit to Genebank submission requirement because it includes contigs less than 200 bp.

#### EPISEQ® CS

*Usability*: Unlike Bugseq^TM^, EPISEQ^**®**^ CS does not support direct import of FASTQ files from BaseSpace or SRA. Users are required to select the organism and sequencing technology prior to uploading the FASTQ files. Once the files are submitted, an “Uploading monitor” page opens to track upload progress. The “Dashboard” displays a visual image of sequencing performance, including resistance gene detection. The “Strain” page is divided into two sections: “Samples” and “Panels.” In the “Samples” section, users can filter by various options including upload date, detected organisms, and navigate to detailed information for each sample to create a panel. The “Panels” section shows related samples grouped within a panel and an organization, along with metadata of all the samples included in that specific panel. Annual standard subscription includes 200 credits redeemable for analysis of 200 samples.

*Analysis features*: With a standard subscription, only Illumina FASTQ files are accepted. The advanced subscription option is needed to analyze ONT Nanopore FASTQ files. EPISEQ® CS only analyzes most clinically related bacterial species, a major limitation. EPISEQ® CS utilizes their own custom curated database combined with public databases to perform sequencing QC, identity check, AMR, and plasmid detection, a major advantage. Whole genome MLST (wgMLST) is available for typing. A “Panel” for epidemiological analysis was created automatically when more than 2 samples are present in a species, and users also can create panels with selected samples. The “epidemiological analysis” page displayed all information including resistome, metadata, virulome along with dendrogram and similarity distance matrix, and users can toggle each section (Figure 1B). A PDF format report includes dendrogram, QC, and typing was generated and downloadable for created panels. Help center displayed related documentation by topic.

#### Solu

*Usability*: Like EPISEQ^**®**^ CS, Solu also does not support direct import of FASTQ files from BaseSpace or SRA. Uploading files was quick and straightforward, with the platform automatically detecting the file input type. Analysis started as soon as the first sample was uploaded, and the main page displayed the real-time progress of the analysis. In general, the analysis results were available in less than 30 minutes. The main page showed a list of all uploaded samples with species, AMR genes, MLST, SNP cluster and plasmid information. Users could also access detailed information for each sample by clicking on the sample name. Annual plan subscription is required and includes 500 ∼ 750 samples. However, if the maximum number of allowed samples is reached, users can delete previously uploaded samples and analyze additional samples.

*Analysis features*: Solu accepted both Illumina and ONT Nanopore FASTQ files. Each sample view included an overview, read quality, assembly quality, AMR genes, MLST assignment, plasmid identification, similar public genomes, comparative analysis, with downloadable files. Uploaded samples were automatically grouped by species, and single nucleotide polymorphism (SNP) clustering analysis was performed. The “Collection” feature allowed users to include or exclude samples for comparative analysis and SNP-based clustering within the selected group. Users could download AMR identification data of the selected samples and SNP distance data in a single Excel file, with each species organized into individual worksheets. The phylogenetic tree could be customized using the “Tree control” function and exported as a .png format file (Figure 1C). The resistome heatmap (Figure 1D) was grouped by species and included AMR gene prediction, match strength, and the predicted gene location (chromosome vs plasmid). The resistome heatmap could be filtered and reordered. Two standout features were the SNP-based “Comparison” across uploaded species and the “Similar Public Genomes” tool, which assessed relatedness using global reference databases. However, the FASTA file format doesn’t fit to Genebank submission requirement because FASTA header has over 50 characters and includes contigs less than 200 bp.

### Performance assessment

#### Species identification

To assess species identification ability, clinically common bacterial species based on the MALDI-TOF identification were selected. However, compared to MALDI-TOF that many clinics adopted for pathogen identification, WGS analysis was able to discriminate more subtly which resulted in the change of species/ subspecies for five isolates (Table 2). When there was discordance in species identification between WGS analysis and MALDI-TOF identifications, we used pubMLST species ID as reference species identification tool. Since EPISEQ® CS’ pathogen identification was limited to certain predetermined species, *K. michiganensis* and *K. quasipenumoniae* were not processed by EPISEQ® CS platform. Additionally, EPISEQ® CS misidentified one *E. coli* isolate (Table 2). Solu correctly identified most (87/90) isolates but failed to discriminate *Enterobacter cloacae* complex and *Serratia spp.* The BugSeq^TM^ metagenomic classification feature properly identified almost all (89/90) isolates, except one *Serratia spp* (Table 2).

**Table 2.** Species identification outcomes of the web-based platforms

Organism (MALDI-TOF)	Organism (WGS, pubMLST)	No. of isolates	BugSeq^TM^	EPISEQ® CS	Solu
*Escherichia coli*	*Escherichia coli*	19	19 *E. coli*	18 *E. coli,* 1 unidentified	19 *E. coli*
*Klebsiella pneumoniae*	*Klebsiella pneumoniae*	19	19 *K. pneumoniae*	19 *K. pneumoniae*	19 *K. pneumoniae*
*Klebsiella pneumoniae* **^#^**	*Klebsiella quasipneumoniae* ^#^	1	*K. quasipneumoniae*	Not Applicable	*K. quasipneumoniae*
*Klebsiella oxytoca*	*Klebsiella oxytoca*	1	*K. oxytoca*	*K. oxytoca*	*K. oxytoca*
*Klebsiella oxytoca* **^#^**	*Klebsiella michiganesis* ^#^	1	*K. michiganesis*	Not applicable	*K. michiganesis*
*Klebsiella aerogenes*	*Klebsiella aerogenes*	1	*K. aerogenes*	*K. aerogenes*	*K. aerogenes*
*Pseudomonas aeruginosa*	*Pseudomonas aeruginosa*	19	19 *P. aeruginosa*	19 *P. aeruginosa*	19 *P. aeruginosa*
*Staphylococcus aureus*	*Staphylococcus aureus*	15	15 *S. aureus*	15 *S. aureus*	15 *S. aureus*
*Enterococcus faecalis*	*Enterococcus faecalis*	7	7 *E. Faecalis*	7 *E. Faecalis*	7 *E. Faecalis*
*Acinetobacter baumannii*	*Acinetobacter baumannii*	2	2 *A. baumannii*	2 *A. baumannii*	2 *A. baumannii*
*Acinetobacter lwoffii*	*Acinetobacter lwoffii*	1	*A. lwoffii*	Not applicable	*A. lwoffii*
*Enterobacter cloacae* **^#^**	*Enterobacter kobei* ^#^	1	*Enterobacter kobei*	*Enterobacter cloacae*	*Enterobacter cloacae*
*Enterococcus faecium*	*Enterococcus faecium*	1	*E. faecium*	*E. faecium*	*E. faecium*
*Serratia marcescens* **^#^**	*Serratia ureilytica* ^#^	1	*S. ureilytica*	*S. marcescens*	*S. marcescens*
*Serratia marcescens* **^#^**	*Serratia sarumanii* ^#^	1	*Serratia sp*.	*S. marcescens*	*S. marcescens*

# indicates mismatched species identification between MALDI-TOF and WGS.

#### Multi-locus sequence typing

Three platforms used different sequence typing schemas: For *Escherichia coli*, BugSeq^TM^ used Pasteur and Achtman, EPISEQ® CS used Pasteur and Warwick, and Solu used Achtman. For *Serratia sp.,* only BugSeq^TM^ included a typing scheme. Sequence type (ST) assignments were generally consistent for all three platforms. However, the EPISEQ® CS schema occasionally assigned multiple ST for some *Escherichia coli*, *Klebsiella pneumoniae, Pseudomonas aeruginosa, Staphylococcus aureus,* and *Enterococcus faecalis* samples. For example, one *S. aureus* sample was assigned ST30 by BugSeq^TM^ and Solu while EPISEQ® CS assigned multiple STs—6,601, 7,293, 210, 8,130, 30, and several “NA” even though all three used Oxford schema. Similarly, EPISEQ® CS assigned a *K. pneumoniae* sample two ST assignments: 280 and 1,201 while both BugSeq^TM^ and Solu assigned it as ST 280 with the same Pasteur schema. Upon inquiring about the possible reasons for multiple ST assignments in EPISEQ® CS, the product developers revealed that the issue was due to the use of an outdated version of the SPAdes assembler. They confirmed that an updated version was being implemented, which resolves the issue of multiple ST assignment for a single isolate.

#### AMR gene detection

**Figure 2.**
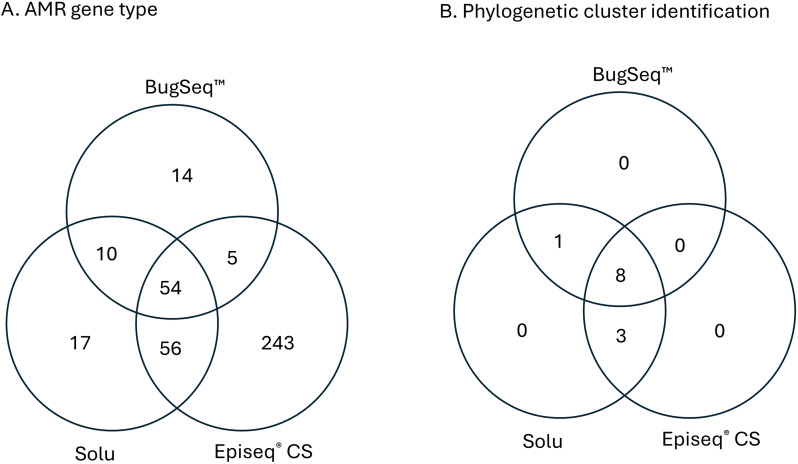
Overlap number of detected antimicrobial resistance gene types (A) and epidemiological clusters (B) among 90 isolates across three microbial WGS analysis platforms. A. The Venn diagram shows 54 common AMR gene types shared among three platforms. B. The Venn diagram shows 8 clusters commonly detected across three platforms.

The number of detectable AMR genes varied across the three platforms. EPISEQ® CS detected a total of 358 types of AMR genes and point mutations among the 90 isolates, and Solu and BugSeq^TM^ detected 137 and 83, respectively (Table 3) but all three platforms detected 54 types of AMR genes (Figure 2A). The high number in EPISEQ® CS is due to multiple gene variants call for a single open reading frame when gene identity was below 100%. As a result, genes such as *bla*OXA-396, *bla*OXA-494, and *bla*OXA-904 were identified exclusively by EPISEQ® CS in certain isolates. Compared to EPISEQ® CS, BugSeq^TM^ was stringent in calling AMR gene detection.

**Table 3.** Performance summary

Performance	BugSeq^TM^	EPISEQ® CS	Solu
Species identification	Identify 89/90 isolate’s species correctly *Serratia* schema	Identify 83/90 isolate’s species correctly Multiple MLST typing	Identify 87/90 isolate’s species correctly
AMR gene and point mutation detection	Detected 83 AMR gene types and point mutations among 90 isolates	Detected 358 AMR gene types and point mutations among 90 isolates Multiple AMR genes assigned for the same contig region	Detected 137 AMR gene types and pint mutations among 90 isolates
Cluster identification	Identify 9 clusters 4 *Escherichia coli* 2 *K. pneumoniae* 2 *S. aureus* 1 *E. faecalis* 0 *P. aeruginosa*	Identify 11 clusters 3 *Escherichia coli* 3 *K. pneumoniae* 2 *S. aureus* 1 *E. faecalis* 2 *P. aeruginosa*	Identify 12 clusters 4 *Escherichia coli* 3 *K. pneumoniae* 2 *S. aureus* 1 *E. faecalis* 2 *P. aeruginosa*
Overall	Recommend for various species identification from the culture free specimens and quality metrics	Recommend for in depth analysis with bit of bioinformatic knowledge to configure AMR gene and MLST	Recommend for fast turnaround time

#### Phylogenetic cluster identification

Epidemiological relationships among isolates of the same species were calculated using different metrics: BugSeq^TM^ used refMLST for inter genome allele distance, EPISEQ® CS used wgMLST for similarity matrix, and Solu used SNP distance from reference genome. BugSeq™ assigned a seven-digit cluster address based on allele differences calculated with refMLST (reference-based MLST),^8^ with the final digit indicating whether samples were within 5 allele differences. EPISEQ® CS defined clusters based on a default similarity threshold of 99.91% while Solu grouped samples into clusters if they were within 20 SNPs. Among the 90 isolates, all three platforms identified: 8 clusters in *Escherichia coli* (3 clusters), *Klebsiella pneumoniae* (2 clusters), *Staphylococcus* aureus (2 clusters), and *Enterococcus* faecalis (1 cluster). BugSeq™ didn’t identify three clusters: one *K. pneumoniae* cluster and two clusters in *P. aeruginosa* due to greater measured allele distances (Table 3). But BugSeq™ and Solu identified one additional *E. coli* cluster that EPISEQ® CS missed due to a failure in species identification for one *E. coli* sample (Figure 2B). This clustering discrepancy ensued because Bugseq^TM^ utilizes refMLST,^8^ which sometimes can yield higher allele distances compared to cgMLST (core-genome MLST). Additionally, BugSeq™ applies species-specific allele distance thresholds, which may further contribute to differences in clustering outcomes.

## Discussion

Cloud-based WGS analysis platforms, usually hosted by vendors, have several merits: (1) no hardware/software installation (2) no maintenance and updates of hardware, (3) user-friendly with graphic interface, (4) faster turnaround than in-house platforms due to the high computing resources. The main limitation of cloud-based platforms is file uploading time which depends on the internet speed, and users can’t change the pipeline run threshold as all pipelines are run on default setting.

All three platforms were user-friendly, and deep expertise in bioinformatics was not necessary to navigate platforms and access the result output. While there is no WGS analysis platform that serves as gold standard for clinical WGS analysis for microbes, each platform we reviewed here has strengths and weaknesses (Table 3). Bugseq^TM^ specializes in pathogen identification with metagenomic classification pipeline, but its unique cluster address assignment does not always match compared to the other two platforms, especially for the *Pseudomonas*, and number of AMR gene types was stringent. EPISEQ® CS might be ideal for users with some bioinformatics knowledge for interpretation as it sometimes assigned multiple STs to a single isolate and numerous AMR gene names to a single locus. In addition, users need to know isolates’ species prior to a data upload and can’t get the analysis results when the species identity falls outside of limited identifiable species (currently total 14 species). Solu provides fast turnaround but not ideal for subspecies discrimination like *Serratia* or *Enterobacter*. In addition, for GenBank submission of sequence data, extra work is required: FASTA file header should be shortened to meet requirements, and the reads coverage depth will need to be obtained through other sources to accomplish submission process.

Limitation: The three platforms reviewed in this study were selected based on availability through government contracting and approval for trial use at our facility. Other platforms may exist that were either unavailable to us or not eligible for government procurement and, therefore, are not included in this study.

While cloud base platforms offer practical options for integrating WGS into routine clinical and IPC practices, factors such as per-sample pricing, IT infrastructure limitations, data security and privacy requirements, specific use cases, and compatibility with existing informatics systems will ultimately guide users in selecting the most appropriate platform for their needs.

## Supporting information

10.1017/ash.2026.10316.sm001 Hwang et al. supplementary material Hwang et al. supplementary material

## Supplementary material

The supplementary material for this article can be found at https://doi.org/10.1017/ash.2026.10316.
